# Immune-Monitoring Disease Activity in Primary Membranous Nephropathy

**DOI:** 10.3389/fmed.2019.00241

**Published:** 2019-11-08

**Authors:** Paolo Cravedi, Marta Jarque, Andrea Angeletti, Àlex Favà, Chiara Cantarelli, Oriol Bestard

**Affiliations:** ^1^Renal Division, Department of Medicine, Icahn School of Medicine at Mount Sinai, New York, NY, United States; ^2^Experimental Nephrology Laboratory, Biomedical Research Institute of Bellvitge (IDIBELL), Barcelona, Spain; ^3^Nephrology, Dialysis and Renal Transplant Unit, Department of Experimental Diagnostic and Specialty Medicine (DIMES), St. Orsola Hospital, University of Bologna, Bologna, Italy; ^4^UO Nefrologia, Dipartimento di Medicina e Chirurgia, Azienda Ospedaliero-Universitaria Parma, Parma, Italy; ^5^Kidney Transplant Unit, Nephrology Department, Bellvitge University Hospital, Barcelona University, Biomedical Research Institute of Bellvitge (IDIBELL), Barcelona, Spain

**Keywords:** membranous nephropathy, glomerulonephritis recurrence, PLA_2_R, THSD7A, autoreactive B cells

## Abstract

Primary membranous nephropathy (MN) is a glomerular disease mediated by autoreactive antibodies, being the main cause of nephrotic syndrome among adult patients. While the pathogenesis of MN is still controversial, the detection of autoantibodies against two specific glomerular antigens, phospholipase A2 receptor (PLA_2_R) and thrombospondin type 1 domain containing 7A (THSD7A), together with the beneficial effect of therapies targeting B cells, have highlighted the main role of autoreactive B cells driving this renal disease. In fact, the detection of PLA_2_R-specific IgG4 antibodies has resulted in a paradigm shift regarding the diagnosis as well as a better prediction of the progression and recurrence of primary MN. Nevertheless, some patients do not show remission of the nephrotic syndrome or do rapidly recur after immunosuppression withdrawal, regardless the absence of detectable anti-PLA_2_R antibodies, thus highlighting the need of other immune biomarkers for MN risk-stratification. Notably, the exclusive evaluation of circulating antibodies may significantly underestimate the magnitude of the global humoral memory immune response since it may exclude the role of antigen-specific memory B cells. Therefore, the assessment of PLA_2_R-specific B-cell immune responses using novel technologies in a functional manner may provide novel insight on the pathogenic mechanisms of B cells triggering MN as well as refine current immune-risk stratification solely based on circulating autoantibodies.

## Introduction

Primary membranous nephropathy (MN) is an autoantibody-mediated glomerular disease that represents one of the leading causes of nephrotic syndrome in adults (1). MN is characterized by the deposition of anti-podocyte targeted IgG antibodies on the subepithelial layer of the glomerular capillary wall. Autoantibodies deposition leads to the thickening of the glomerular basement membrane, complement activation, and glomerular capillary injury with consequent proteinuria. In ~25% of patients, MN is classified as “secondary,” due to a contemporary detection of a causative disease, such as malignancies, infections, drug reactions, or autoimmune diseases including systemic lupus erythematosus (2, 3). The natural history of the untreated disease is variable: spontaneous complete remission of primary MN is observed in approximately the 30–40% of patients (4, 5), whereas 30% of cases develop end-stage kidney disease (ESKD) generally over 10–15 years (6, 7). In kidney transplant recipients, MN relapses appear in 10–45% of cases (8–12) and occur as a *de novo* disease in about 2% of recipients (13, 14).

Current understanding of MN pathophysiology comes from studies in rodent models. In 1959, Heymann et al. (15) described a model of MN, now defined as active Heymann nephritis, which was induced by immunizing Lewis rats with intraperitoneal injections of crude kidney extracts, together with complete Freund's adjuvant. This resulted in a disease characterized by subepithelial immune complexes similar to human MN. Subsequent *in vivo* and *in vitro* studies have led to a better understanding of how subepithelial immune deposits lead to podocyte injury and proteinuria. Complement-mediated cytotoxicity plays a major role in the disease pathogenesis, especially the terminal complement complex C5b-9 (membrane attack complex—MAC), which is detectable in the urine of patients with MN and considered a marker of podocytes injury (16–20). Data suggest that in primary MN, complement cascade is firstly activated by the mannose binding lectin pathway, leading to the formation of C3 deposits in the subepithelial space along with MAC on podocyte membranes (21–23).

The identification of the cell surface protease neutral endopeptidase (NEP) as a target podocyte autoantigen in a newborn with MN represented a cornerstone in our understanding of MN pathophysiology. Pierre Ronco and Hanna Debiec described the case of a mother genetically deficient in NEP that had given birth to an infant who developed antenatal nephrotic syndrome (24). During the previous pregnancy, the mother generated circulating anti-NEP that crossed the placenta and targeted NEP on the fetal kidney during her subsequent pregnancy, leading to *in situ* immune deposits. Therefore, NEP represents the first podocyte protein demonstrated to be a target antigen in human MN (25).

Identification of autoantibodies reactive against M-type phospholipase A2 receptor type 1 (PLA_2_R) (26) and, later, against thrombospondin type 1 domain containing 7A (THSD7A) (27), two podocyte-expressed proteins, represented a further major step forward in defining the disease pathogenesis. Autoantibodies against such antigens can be detected in the 75–85% of primary MN patients (28, 29): anti-PLA_2_R autoantibodies are present in ~70–80% of adult cases, particularly in men (26, 30), whereas anti-THSD7A antibodies may be detected in only 3–5% of adults with primary MN, mainly in women (27, 31). Only about 1% of MN patients have both anti-PLA_2_R and anti-THSD7A autoantibodies detectable (32).

A 2019 study (33) showed that, in MN patients without detectable anti-PLA_2_R or anti-THSD7A autoantibodies, exostosin1/exostosin2 could represent target antigens. The authors performed mass spectrometry on laser microdissected glomeruli and immunohistochemistry on kidney biopsy of 22 MN patients, including 7 with anti-PLA_2_R antibodies and 15 without, detecting exostosin1/exostosin2 expression uniquely in five cases without detectable circulating anti-PLA_2_R antibodies. In a larger cohort of 209 MN patients negative for circulating anti-PLA_2_R antibodies, immunohistochemistry revealed bright granular glomerular basement membrane staining for exostosin 1/exostosin 2 in 16 cases (33). Eleven of the 16 cases showed signs of lupus nephritis or autoimmunity, suggesting that exostosin 1/exostosin 2 may represent a potential marker of a specific subtype of MN, most commonly associated with autoimmune diseases (33).

Altogether, these mechanistic findings have highlighted the key role of B cells in the pathogenesis of MN, both as autoantibody producing cells (34) and as antigen presenting cells (35), thus providing the basis for B-cell target therapies (36–39). However, response to such therapies remains unpredictable and the identification of subjects who would develop spontaneous remission (in whom immunosuppression could be avoided) is still very challenging. The discovery of MN-specific antigens has allowed the development of many diagnostic and prognostic serologic tests and optimal non-invasive biomarkers for monitoring disease activity. Nevertheless, while the assessment of autoantibodies provides useful information about the humoral memory immune response, other assays are needed to better immune-risk stratify patients and to tailor treatment in a personalized fashion.

## Current Clinical MN Biomarkers: Serum Creatinine, Urinary Protein and Kidney Biopsy

According to the most recent *Controversies Conference* on KDIGO guidelines (39), proteinuria, and serum creatinine are still considered the gold-standard biomarkers to risk-stratify MN patients. For instance, individuals with subnephrotic proteinuria have excellent long-term renal survival, therefore, immunosuppression is not recommended (39). Conversely, in patients with proteinuria above 4–5 g/24 h, MN prognosis may range from spontaneous remission to development of ESKD.

Urinary markers of renal tubular damage, such as, Beta2 microglobulin, N-acetyl-β-D-glucosaminidase (NAG) and retinol-binding protein (RBP), kidney injury molecule 1 (KIM-1) and neutrophil gelatinase-associated lipocalin (NGAL) have been also proposed to risk-stratify patients with MN. Yet, the levels of these biomarkers seem to not correlate with the severity of the disease (40).

Despite its invasive nature, kidney biopsy is still important for the diagnosis of MN, in particular among patients with altered kidney function and evidence of possible secondary causes (41), but the capacity of histological lesions to predict outcomes or response to therapy is limited at best. Hence, new approaches to better risk-stratify MN patients are highly needed in the clinical setting.

## Target Antigens in MN

Over the last decade, discovery of target podocyte antigens and the development of commercial assays for the detection of serum anti-PLA_2_R and anti-THSD7A autoantibodies has revolutionized the traditional algorithms for diagnosis and management of MN, particularly due to their high specificity for disease diagnosis (26, 27). Such autoreactive antibodies recognize the target conformational epitopes on the membrane protein expressed on glomerular podocytes under non-reducing conditions and are predominantly of the IgG4 subclass. Importantly, both autoantibodies are emerging as clinical biomarkers to predict outcome in MN patients.

### Thrombospondin Type 1 Domain Containing 7A (THSD7A)

THSD7A is a large transmembrane glycoprotein expressed by podocytes. In Europe and United States only 3% of MN subjects expresses anti-THSD7A autoantibodies (predominantly IgG4), while it increases to a 9% in Japan (27, 31, 42, 43). Importantly, anti-THSD7A antibodies induce a MN-like pattern of disease when injected in mice (29). In a recent retrospective study, Zaghrini et al. (44) developed a new ELISA assay to detect THSD7A-specific antibodies: levels of anti-THSD7A autoantibodies correlated with disease activity and with response to treatment. Also, patients with high titers at baseline had a poorer clinical outcome. I has also been reported an association between anti-THSD7A autoantibodies and malignancies (42, 43, 45), but this needs to be better clarified in larger, multicenter studies.

### Phospholipase A2 Receptor Type 1 (PLA_2_R)

The M-type phospholipase A2 receptor (PLA_2_R) is one of four members of the mannose receptor in mammals (46). PLA_2_R is a multifunctional receptor for soluble phospholipase A2 (sPLA2), which is described as a pro-inflammatory enzyme and PLA_2_R acts as a scavenger receptor to remove secreted PLA2 enzyme (47). Despite this receptor being highly expressed by human podocytes as well as by neutrophils and alveolar type II epithelial cells (26, 48, 49), autoantibodies against PLA_2_R exclusively induce nephrotic syndrome without apparent impairment in other organs.

The complexity of the PLA_2_R structure is illustrated by the identification of distinct immunogenic PLA_2_R epitopes, including a cysteine-rich domain (CysR), a fibronectin type II domain and eight distinct C-type lectin domains (CTLD1–8) (50), which are dependent on the protein conformation (26). Main antigenic epitopes recognized by anti-PLA_2_R antibodies have been recently identified and reported to be sensitive to reducing agents, thus confirming that conformational structure is of great importance in PLA_2_R epitopes (51, 52). A further dominant epitope of PLA_2_R (P28mer) was recently identified being also a dominant epitope of THSD7A in the N-terminal domain, suggesting that this shared motif could be involved in the initial B-cell activation in MN (53).

## Genetic Susceptibility and Humoral Autoimmune Response in MN

A genetic predisposition for MN was initially speculated by the associative evidence linking variants in the HLA *locus* and the risk of developing MN (54). Years later, family case reports of MN were also described (55).

Several genome-wide association studies (GWAS) have recently associated risk alleles in HLA genes with the increase risk of MN. Stanescu et al. (56) defined the association between HLA-DQA1 allele with MN in Caucasian individuals, suggesting that the interaction between sequence variations in immune-proteins and glomerular components may explain a trigger-target model in the disease development. Such interaction between PLA_2_R and HLA-DQA1 variants was also studied in an Asian cohort with similar results (57). More studies confirmed this association in different cohorts of MN patients (58–61), but the related mechanisms remain unknown.

The possible role of specific HLA alleles in MN was further investigated in two recent studies. Cui et al. (62) genotyped HLA-DRB1, DQA1, DQB1, and DPB1 genes in 261 primary MN patients and in 599 healthy controls. These investigators confirmed that risk alleles of HLA-DQA1 and PLA_2_R are significantly associated with the susceptibility to MN. Particularly, authors showed that these risk alleles are associated with the presence of circulating anti-PLA_2_R antibodies as well as to the increased expression of PLA_2_R in the glomeruli. Authors also detected the classical DRB1^*^1501 and DRB1^*^0301 alleles, showing significant independent effects on the risk of MN among the ethnic group of *Han Chinese*. Le et al. (63) sequenced HLA locus in 99 anti-PLA_2_R-positive MN subjects and in 100 healthy controls. Again, the association between DRB1^*^1501 and anti-PLA_2_R positive MN was demonstrated, and suggested DRB3^*^0202 as new risk allele for MN. These two alleles were subsequently confirmed in an independent cohort of 285 controls and 293 cases. Although DRB1^*^1502 was not revealed as a risk allele for MN, it was associated with significantly higher levels of anti-PLA_2_R autoantibodies and a significantly increased risk of progression to ESKD (64).

Altogether, GWAS has provided robust data about the genetic susceptibility to MN, suggesting that genetic tests could become a non-invasive tool to risk-stratify MN patients (65), although more data testing these associations in different ethnic groups are needed (66).

## Immune-monitoring of Autoreactive Antibodies

### Detection of PLA_2_R Antigen in the Kidney

Anti-PLA_2_R IgG4 autoantibodies are detected in the sub-epithelial immune deposits using immunofluorescence or immunohistochemistry in patients with primary MN (67). In normal kidneys or other glomerular diseases, the PLA_2_R antigen is weakly expressed on the podocyte surface (67). Generally, a strong association between glomerular PLA_2_R staining and circulating anti-PLA_2_R antibodies is found (28, 60, 68), particularly when autoantibody levels are measured at the time of the biopsy assessment (69). However, glomerular PLA_2_R staining is not considered a diagnostic test for active disease, since the positivity of glomerular PLA_2_R staining with undetectable circulating anti-PLA_2_R autoantibodies is unlikely (28, 69, 70) and may reflect an immunologically inactive disease as a positive PLA_2_R antigen can persist for weeks or months after remission (67).

### Detection of Serum Anti-PLA_2_R Autoantibodies as a Diagnostic Tool

Western blotting was initially performed to detect anti-PLA_2_R (26) and anti-THSD7A (27) autoantibodies, but this test is inadequate for routine clinical use. The first commercially available assay for serum anti-PLA_2_R autoantibodies detection was an indirect immunofluorescence assay (CBA-IFA; Euroimmun, Luebeck, Germany), based on a semi-quantitative determination, and therefore, not ideal for monitoring therapeutic response and disease progression. Most clinical laboratories routinely use an ELISA-based assay (Euroimmun), because it is able to quantify anti-PLA_2_R autoantibodies, but this assay is not as sensitive as CBA-IFA assays. Conversely, the CBA-IFA anti-PLA_2_R immunoassays detection may be considered only when diagnosis of PLA_2_R-associated MN is strongly suspected, but there is a negative ELISA test. The most recent diagnostic assay is a laser bead immunoassay (ALBIA; Mitogen Advanced Diagnostics Laboratory, Calgary, Canada), that allows a sensitive and a quantitative detection of these autoantibodies. This assay allows the detection of different molecules such as antibodies, complement or cytokines. A comparison between the CBA-IFA, ELISA and ALBIA platforms, showed similar capacity across the different tests to detect anti-PLA_2_R autoantibodies (71).

### Serum Anti-PLA_2_R Autoantibodies as a Risk-Prognostic Biomarker of MN

Different groups have suggested the use of anti-PLA_2_R autoantibodies to predict spontaneous remission of MN. Hofstra et al. (72) reported that spontaneous remission is inversely related to high antibodies titers measured by up to 6 months after biopsy assessment. Similarly, Timmermans et al. (73) showed that, among 109 MN patients, subjects with detectable serum anti-PLA_2_R autoantibodies at the time of biopsy had a lower probability for spontaneous remission than seronegative patients. In a retrospective study including 68 patients with biopsy-proven MN, Jullien et al. (74), reported that spontaneous remission was correlated with low titers of anti-PLA_2_R autoantibody at time of biopsy. These data were recently confirmed by a prospective study involving 62 MN patients: complete spontaneous remission was more common in subjects with lower anti-PLA_2_R autoantibody levels at the time of diagnosis (<40 UI/mL) (75).

Beck et al. (76) evaluated the relationship between changes in serum PLA_2_R-specific autoantibodies levels and the response to B cell-depleting antibody rituximab therapy in 35 adult patients with MN. Circulating autoantibodies were detected in 71% of patients at baseline and levels decreased after rituximab therapy in the majority of them. The reduction of anti-PLA_2_R autoantibody levels anticipated the decline of proteinuria, and in one particular patient with a relapse of proteinuria, the reappearance of the autoantibody in serum preceded the recurrence of MN. However, proteinuria may persist, regardless the presence of autoreactive anti-PLA_2_R antibodies due to irreversible capillary wall injury thus, perpetuating albuminuria levels in absence of active autoimmunity.

More recently, Ruggenenti et al. (77) investigated the association between treatment effect, circulating anti-PLA_2_R autoantibodies and genetic polymorphisms predisposing to antibody production in 132 MN patients with nephrotic range proteinuria treated with rituximab. Outcome of patients with or without detectable anti-PLA_2_R autoantibodies at baseline were similar. However, among 81 patients with autoantibodies, lower anti-PLA_2_R autoantibodies titer at baseline and full depletion at 6 months post-treatment strongly predicted remission over a median follow-up period of 30.8 months. All 25 patients displaying complete remission were preceded by undetected anti-PLA_2_R autoantibodies in circulation, while re-emergence of circulating antibodies predicted clinical disease relapse. Accordingly, a further study involving 30 patients with MN and elevated anti-PLA_2_R autoantibodies (78) showed that clinical remission was heralded by a reduction in circulating autoantibodies.

Collectively, the above studies and further published data (79–83) suggest that serial measurements of anti-PLA_2_R autoantibody titers in the serum may help at risk-stratifying patients, allowing to personalize treatment and to reduce the side-effects related to over-immunosuppression.

However, antigen-specific memory B cells may exist and be ready to develop a rapid and effective secondary immune response even in absence of detectable circulating autoantibodies. This suggests that the assessment of the humoral auto-immune response using other cell-based assays may significantly improve the understanding of the effector mechanisms of the disease in patients with primary MN.

### PLA_2_R Epitope Spreading and Disease Progression

Epitope spreading is a common immunopathogenic response to self-antigens: the immune response primary involves the so-defined immunodominant epitope recognized by most autoantibodies, then expands to the intramolecular epitope on the same protein (intramolecular epitope spreading) or to dominant epitopes on neighboring molecules (intermolecular epitope spreading) (84, 85). The result is an increased diversity in antibody repertoire, leading to a broader overall immune response. Epitope spreading for the CysR epitope of PLA_2_R has been recognized as independent risk factor for reduced renal survival (86). In the GEMRITUX (Evaluate Rituximab Treatment for Idiopathic Membranous Nephropathy) randomized controlled trial (87), including a cohort of 58 patients positive for anti-PLA_2_R-specific autoantibodies randomly treated with rituximab or conservative therapy, epitope spreading strongly correlated with serum titer of anti-PLA_2_R autoantibodies The absence of epitope spreading at onset was an independent predictor of remission at 6 months and at last follow-up (median of 23 months) (88). Of interest, 10 of the 17 patients who had epitope spreading at baseline and were treated with rituximab, showed reversal of epitope spreading at 6 months (88). The anti-PLA_2_R autoantibody titer has been shown to correlate with the degree of epitope spreading (88). Therefore, due to the lack of epitope-specific assays for anti-PLA_2_R autoantibodies for clinical practice, the total titer of anti-PLA_2_R autoantibodies could be considered a surrogate of epitope spreading (88).

### Immune Cell Phenotyping and Circulating Cytokines in MN

#### Non-antigen-specific Cell Subset Measurements

A few studies have investigated the immune phenotype of MN patients and its changes in relation to treatment (Table 1). Some investigators reported an increase of the CD4^+^/CD8^+^ T cell ratio in MN patients with or without nephrotic proteinuria (89, 90). Some evidence has shown a reduction of CD8^+^ T cells in patients with MN and nephrotic syndrome when compared to healthy subjects (91). This broad phenotype seems to be associated with a more favorable prognostic response to classical immunosuppressive therapy (92), but not to anti-CD20 depletion (93). MN is characterized by a predominance of IgG4 subclass autoantibodies, thus suggesting an involvement of a Th2 immune response, which has been described in some series (94–96).

**Table 1:** Studies on the immune phenotype of patients with membranous nephropathy.

**Reference**	**Patients' characteristics (number)**	**Assay/biomarkers**	**Results**
Ozaki et al. (89)	MN (30): - Untreated, - with Prednisolone, - incomplete remission, - complete remission	Flow cytometry/ Helper, suppressor T cells	• Untreated nephrotic patients showed a significant decreased in suppressor T cell levels and a relative increase in helper T cells. • Prednisolone-treated patients showed an increased number of suppressor T cells.
Wang et al. (90)	MN (66): - No previous IS HC (40)	Flow cytometry/ Treg, B and T cells	• Treg cells were decreased in MN patients. • B cells were increased in MN patients. T cells (CD4^+^/CD8^+^) were increased in MN patients. • No association between circulating B cells and disease activity.
Cagnoli et al. (91)	MN (27) - 12/27 nephrotic syndrome, - 6/27 isolated proteinuria, - 9/27 complete remission - No previous IS MCD (20) IgAN (12) HC (15)	Indirect IF/ Total peripheral T cells, CD4^+^, and CD8^+^ T cells	• Patients with MN and nephrotic syndrome presented a CD4^+^/CD8^+^ ratio greater than the control group due to a reduction of CD8^+^ T cell subset.
Zucchelli et al. (92)	MN (39): - 23/39 were treated with methylprednisolone + chlorambucil - 16/39 not treated - Patients with serum creatinine >1.7 mg/dl were excluded HC (30)	Indirect IF/ Total peripheral T cells (LEU4), helper T cells (LEU3a), cytotoxic T cells (LEU2a)	• Helper/cytotoxic T cell ratio was significantly higher at baseline in MN patients than the in controls due to a reduction of LEU2 cell subset. • Baseline helper/cytotoxic T cell ratio was significantly higher in patients achieving remission as compared to non-responder patients.
Taube et al. (93)	MN (21) MCD (11) FSGS (15)	Suppressor cell function evaluation due to response to Concanavalin A	• Significant reduction in lymphocyte transformation in each group of patients as compared to the control group. • Suppressor cell function was decreased in each group of patients as compared to the control group.
Hirayama et al. (94)	MN (8): - Proteinuria ranging from 2 to 7 g/day - Creatinine clearance> 100 ml/min/1.73 m^2^ HC (23)	Intracellular cytokine assay by flow cytometry/T-helper cells, Th1 and Th2 cytokines	• Percentages of IL-2+CD4^+^ T cells were significantly lower in MN patients than in the controls. • No differences in percentages of IFN-γ^+^ IL-4^+^CD4^+^ T cells were observed between different groups. • Percentages of IL-10^+^CD4^+^ T cells were significantly higher in MN patients than in the control group.
Masutani et al. (95)	MN (24) MCD (13) FSGS (12) HC (51)	Intracellular cytokine assay by flow cytometry/ T-helper cells, Th1 and Th2 cytokines	• Percentages of IL-4 in MN patients were significantly higher than in the other groups. • Th1/Th2 ratio was significantly lower in MN patients than in the other groups. • Percentages of IL-4 correlated with the amount of proteinuria in MN patients.
Kuroki et al. (96)	MN (14) HC (14)	Flow cytometry/ T cells, T-helper cells, T-cytotoxic cells, B cells Real-time PCR/ Th1 and Th2 cytokines	• CD4/CD8 cell ratio was higher in MN patients than in the control group, although numbers of T and B cells were similar to the control group. • IL-10 and IL-13 mRNA expression levels was higher in MN patients. • IL-4 enhances *in vitro* production of IgG4 by B cells in MN.
Fervenza et al. (97)	MN (20) - Patients were all treated with Rituximab - Creatinine clearance ≥30 ml/min/1.73 m^2^ - Persistent proteinuria >5 g/24 h	Flow cytometry/ T, B and NK cells	• After rituximab treatment, proteinuria decreased and creatinine clearance increased. • None of the T-reg subset analyses showed significant quantitative differences. • Baseline quantification of lymphocyte subpopulations did not predict response to rituximab therapy.
Roccatello et al. (98)	MN (17) - Patients were all treated with rituximab	Flow cytometry/B, T, Treg cells ELISA assay/IL-35 and PLA_2_R antibodies	• After rituximab treatment, proteinuria decreased and serum creatinine remained stable during the follow-up. • Treg percentages were significantly higher after treatment as compared to baseline.
Rosenzwajget al. (99)	MN (25): - 16/25 were treated with NIAT + rituximab - 9/25 were treated with NIAT alone HC (27)	Flow cytometry/ B, T, NK, Treg, γδ-T cellsMultiplex to detect several cytokines/ chemokines	• Percentages of switched (IgD^−^CD27^+^) and non-switched (IgD^+^CD27^+^) memory B cells were higher in MN patients due to a higher percentage of naïve B cells at baseline. • Treg percentages were lower in MN patient at baseline. • After rituximab treatment, responder patients to treatment showed a significantly increased percentage of Treg cells than non-responders.

*FSGS, focal segmental glomerulosclerosis; IFN, interferon; HC, healthy controls; IL, interleukin; IF, immunofluorescence; IS, immunosuppression; MCD, minimal change disease; MN, membranous nephropathy; NIAT, nonimmunosuppressive antiproteinuric treatment; NK, natural killer; Treg, regulatory T cells*.

Interestingly, despite the well-reported role of regulatory T-cells (Treg) in autoimmune diseases (100, 101), limited studies have investigated the role and impact of Tregs in primary MN, with controversial results (97, 98). Recently, Rosenzwajg et al. (99) measured 33 lymphocyte subpopulations and also 27 serum cytokines/chemokines in 25 MN patients and 27 healthy subjects at the time of biopsy. After rituximab treatment, responder patients to treatment showed a significantly increased percentage of Tregs than non-responders concluding that monitoring T-cell subset could be a potential biomarker of MN activity.

#### Cellular Assays Measuring Antigen-Specific Immune Responses

**Figure 1:**
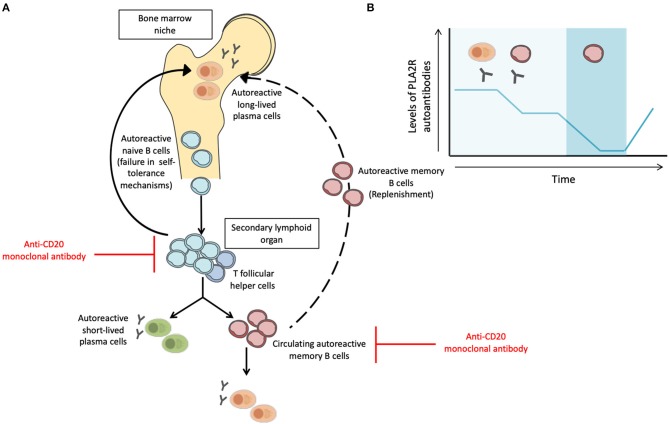
Activation and inhibition of autoimmune B cell responses and the influence of different B cell subsets in the fluctuation of circulating anti-PLA_2_R antibodies. **(A)** After failure in self-tolerance mechanisms, autoreactive naïve B cells may encounter the self-antigen and can be activated in the secondary lymphoid organ by helper signals from T follicular helper cells. Then, B cells can differentiate into short-lived plasma cells, which secrete mainly IgM antibodies or differentiate into memory B cells or long-lived plasma cells after somatic hypermutation and immunoglobulin isotype class switching in the germinal center. After a re-encounter with the self-antigen, memory B cells can rapidly differentiate into antibody-secreting cells, sustaining long-lasting humoral immunity. Memory B cells may occupy empty bone marrow niches after secondary activation replenishing plasma cell pool. Anti-CD20 monoclonal antibodies (Rituximab) mainly target naïve B cells and memory B cells but not long-lived plasma cells. **(B)** Levels of anti-PLA_2_R autoantibodies may fluctuate over time and may become undetectable without indicating MN remission. Memory B cells can be detected in the absence of antibody levels in serum and its rapid differentiation and production of antibodies can be of great importance for a subsequent humoral response. Such effective and rapid response of the memory-B cell population indicates that although anti-PLA_2_R autoantibodies may not be detected in serum, PLA_2_R-specific memory B cells can be a target indicator of MN relapse.

The discovery of anti-PLA_2_R and anti-THSD7A autoantibodies represented a paradigm shift for the diagnosis and management of MN patients. Taking into account the putative pathogenic role of anti-PLA_2_R autoantibodies and the efficacy of B cell depleting therapies (77, 102–104), it is reasonable to speculate that autoreactive memory B cells play a fundamental pathogenic role in MN by fueling a persistent IgG4-specific humoral immune response. However, levels of anti-PLA_2_R autoantibodies fluctuate over time despite persistent renal injury, suggesting that the evaluation of anti-PLA_2_R autoantibodies alone may not capture the global humoral immune response taking place in patients with primary MN (69, 79–81). Once B cells recognize the target antigen through the help of autoreactive T Follicular Helper (T_FH_) cells, B cells can differentiate into short-lived plasmablasts (secreting manly low-affinity IgM antibodies) or into memory B cells (mBC) and long-lived plasma cells after undergoing somatic hypermutation and immunoglobulin isotype class switching in the germinal center. In case of persistence of the priming antigen and T-cell help, auto-reactive mBC can rapidly differentiate into antibody-secreting cells and produce the effector antibodies against the specific target antigen and may finally occupy empty bone marrow niches after secondary activation replenishing plasma cell pool (105, 106). Noteworthy, autoreactive memory B cells can be detected in absence of autoantibody levels in serum and its rapid differentiation and production of antibodies can be of great importance for a subsequent humoral response (Figure 1) (107, 108). Recent works in kidney transplantation have shown the value of measuring circulating allospecific mBC in a functional manner, especially in the absence of detectable alloantibodies in the serum (109–111).

**Figure 2:**
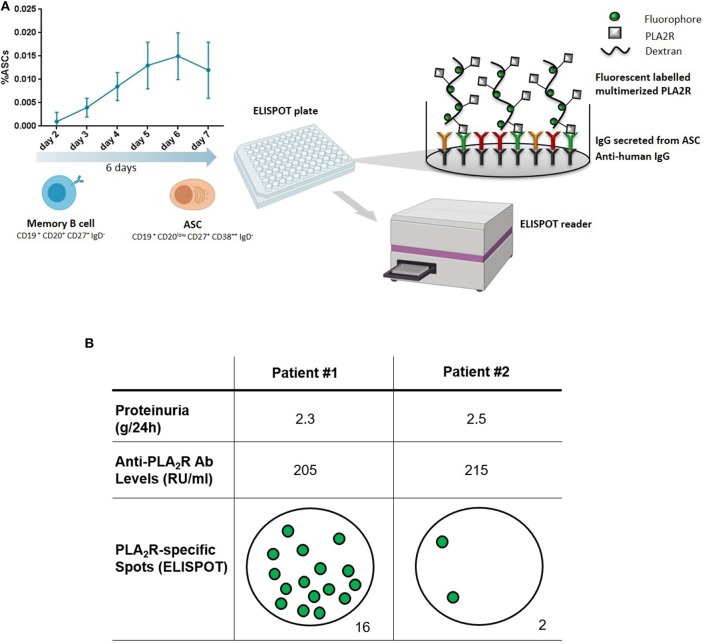
Measuring anti-PLA_2_R reactive memory B cells. **(A)** Peripheral blood mononuclear cells are first polyclonally activated for 6 days to expand the pool of memory B cells and antibody secreting cells (ASC). Expanded cells are next used for an enzyme-linked immune absorbent spot (ELISPOT) assay to detect cells producing antibodies against PLA_2_R. **(B)** Two representative patients with membranous nephropathy and similar levels of proteinuria and circulating anti-PLA_2_R antibodies. Patient #1 has a positive ELISPOT, indicating the presence of autoreactive memory B cells (sign of active disease), while Patient #2 has no detectable autoreactive memory B cells (indicative of a remission phase). Adapted from Luque et al. (112).

Starting from this background, our group has recently developed a new approach to functionally evaluate the PLA_2_R-specific mBC response in MN patients. Using a PLA_2_R-specific B-cell ELISPOT-based immune assay, we have been able to accurately detect circulating mBC capable of producing anti-PLA_2_R-specific antibodies at the time of the flare of disease activity, thus confirming the presence of an active humoral immune response (personal communication). While evaluating PLA_2_R-specific antibody-secreting cell frequencies using an ELISPOT-based assay allows for an accurate detection of mBC responses at the single cell level after a polyclonal mBC culture stimulation, anti-PLA_2_R-specific antibodies may also be detected from these cell culture supernatants using single-antigen beads immune assay. Figure 2 shows two representative patients with similar proteinuria and anti-PLA_2_R autoantibody levels. While the first patient with detectable autoreactive mBC is having a disease flare, the second one has no detectable autoreactive mBC and is therefore predicted to undergo remission. If properly validated, this assay may be used to differentiate patients for whom therapy is needed vs. those who will undergo spontaneous remission.

## Conclusions

Primary MN is the main cause of nephrotic syndrome in adults and is caused by the formation of autoimmune complexes in the glomeruli. Since the identification of different podocyte antigenic targets, the diagnostic strategies and treatment options for MN have significantly improved. The efficacy of rituximab treatment in MN patients has highlighted the importance of B cells in the pathogenesis of the disease (113); therefore a more accurate investigation of autoreactive mBC using new technology may refine current immune-monitoring largely based on the measurement of circulating anti-PLA_2_R or anti-THSD7A autoantibodies.

## Author Contributions

PC, MJ, AA, ÀF, CC, and OB conceived the article contents, prepared the manuscript, and endorsed the final draft submitted.

### Conflict of Interest

The authors declare that the research was conducted in the absence of any commercial or financial relationships that could be construed as a potential conflict of interest.
